# Metal Artifact Reduction in Dental CBCT Images Using Direct Sinogram Correction Combined with Metal Path-Length Weighting

**DOI:** 10.3390/s23031288

**Published:** 2023-01-23

**Authors:** Mohamed A. A. Hegazy, Myung Hye Cho, Min Hyoung Cho, Soo Yeol Lee

**Affiliations:** 1 R&D Center, Ray, Seongnam-si 13494, Republic of Korea; 2 Department of Biomedical Engineering, Kyung Hee University, Yongin-si 17104, Republic of Korea

**Keywords:** metal artifact reduction, beam hardening, direct sinogram correction, dental CBCT, metal segmentation

## Abstract

Metal artifacts in dental computed tomography (CT) images, caused by highly X-ray absorbing objects, such as dental implants or crowns, often more severely compromise image readability than in medical CT images. Since lower tube voltages are used for dental CTs in spite of the more frequent presence of metallic objects in the patient, metal artifacts appear more severely in dental CT images, and the artifacts often persist even after metal artifact correction. The direct sinogram correction (DSC) method, which directly corrects the sinogram using the mapping function derived by minimizing the sinogram inconsistency, works well in the case of mild metal artifacts, but it often fails to correct severe metal artifacts. We propose a modified DSC method to reduce severe metal artifacts, and we have tested it on human dental images. We first segment the metallic objects in the CT image, and then we forward-project the segmented metal mask to identify the metal traces in the projection data with computing the metal path length for the rays penetrating the metal mask. In the sinogram correction with the DSC mapping function, we apply the weighting proportional to the metal path length. We have applied the proposed method to the phantom and patient images taken at the X-ray tube voltage of 90 kVp. We observed that the proposed method outperforms the original DSC method when metal artifacts were severe. However, we need further extensive studies to verify the proposed method for various CT scan conditions with many more patient images.

## 1. Introduction

Dental X-ray cone beam computed tomography (CBCT) systems are now widely used in dental clinics since they can provide precise 3D anatomical images of teeth and upper/lower jawbones, despite their much smaller cost and space requirement as compared to a conventional medical CT system [1]. The precise 3D images can help provide better dental treatments, such as dental implants and orthodontics. As compared to head and body images, dental CT images are more prone to metal artifacts since the patient is likely to have many dental implants, crowns, and dental fillings made of highly X-ray absorbing materials. Dental 3D CT images can be used to generate surface-rendered tooth and bone images for full digital dentistry [2,3]. However, metal artifacts often make big distortions in the surface-rendered images, which could induce big errors in the STL data generation.

Metallic materials used for dental treatment have high physical densities and atomic numbers, hence they can cause severe beam hardening, scattering, and photon starvation during projection data acquisition. Beam hardening and photon starvation make metal artifacts, usually in the form of artificial streaks and dark shadings around metallic objects [4,5,6]. Since CT image reconstruction is based on the assumption that the X-rays are linearly attenuating in the human body, any nonlinear X-ray attenuation behaviors result in artifacts in the reconstructed CT images. Despite many developments in CT technology in the past decades, the reduction of metal artifacts is still a big challenge, especially in dental CT imaging [7]. Various metal artifact reduction (MAR) methods were developed, and those are usually categorized into five methods: the physical modeling-based method, the projection completion-based method, the dual energy-based method, the iterative reconstruction-based method, and the deep learning-based method.

The projection completion-based method deals with the metal projection data as missing data that should be replaced with other projection data. Some methods replace the projection data on metal traces with ones calculated by interpolating the neighboring projection data [8,9,10,11]. Other methods replace the missing projection data with ones generated from the forward projection of a prior image generated from the CT image [12,13,14,15,16]. In the normalized metal artifact reduction (NMAR) method, prior images are generated to produce synthetic projection data that are to be used to normalize the projection data [17,18]. The dual energy-based method aims to reduce the beam hardening artifact by acquiring the projection data at two different X-ray voltage settings to estimate the mono-energetic projection data [19,20,21]. The dual energy-based method works well in reducing the metal artifact, but it may increase the X-ray dose to the patient. The iterative reconstruction-based method compares the forward projected data with the original projection data, and this process is iterated until the error between the two projection data is minimized under some artifact-reducing constraints [22,23,24,25]. The iterative reconstruction-based method has a limitation of excessively long computation time for 3D dental images. Recently, many deep learning-based MAR methods have been proposed. Some methods use deep learning for accurate metal segmentation to improve the performance of the projection completion-based method [26,27]. Some methods try to reduce the metal artifact directly from the CT image by learning from the artifact-free images [28,29,30], while other methods apply deep learning to the projection data [31,32] or combine the benefits of using both domains learning [33]. A huge number of patient images, both artifact-free and artifact-corrupted, are necessary to properly train the deep learning network, which currently limits the use of deep learning for dental CT images.

The physical modeling-based method uses mathematical models for the metal artifact formation by the beam hardening of the polychromatic X-ray beam. This kind of correction method is usually applied to the projection data before the image reconstruction [34,35,36]. One example is the direct sinogram correction (DSC) method introduced by Lee et al. [36]. The DSC method reduces the discrepancy between the measured projection data and the ideal projection data, i.e., the Radon transform of the attenuation coefficient map. They introduced a mapping function that changes the projection data to another in a way that the changed projection data better satisfy the Radon transform. They reported that the DSC method works well in the cases of phantom images and mathematically generated human images [36], but we found that the DSC method fails to correct heavy metal artifacts frequently appearing in clinical dental CT images. The same group proposed another method that uses deep learning as a second stage correction to overcome the limitations of the DSC method [37]. Yet, the new method needs a huge number of patient images, both artifact-free and artifact-corrupted, which were not available in the public domain until now. We propose a modified DSC method that uses the metal path-length information to improve the MAR performance of the DSC method in patient images.

## 2. Methods

### 2.1. Limitations of the Direct Sinogram Correction Method

To reduce metal artifacts in dental CT images, we use the concept of the direct sinogram correction (DSC) method [36,37]. The key idea of the DSC method is mapping the original projection data $P_{0}$, i.e., the measured projection data along a projection line, to a new projection data $P_{c}$ in such a way that the new projection data set better satisfy the Radon transform. Beam hardening of the polychromatic X-ray beam makes the projection data deviate from the true line integral of the X-ray attenuation coefficients, hence the measured projection data set is not equal to the Radon transform of the attenuation coefficient map. Lee et al. [36] made a mathematical model for the beam hardening effect on the projection data considering the photoelectric effect and Compton scattering. From the rigorous mathematical derivations, they found the mapping function ψ that converts the original projection data to a new one in such a way that the new projection data set best satisfies the objective function related to the Radon transform. Once the mapping function has been found, the beam hardening correction can be made by simply mapping all the projection data to new ones before the image reconstruction. Lee et al. [36] have established a mapping function $\psi_{\lambda ,t_{*}}$ to map the original projection data ($P_{0}$) to a corrected projection data ($P_{c}$), as follows:

(1) $$P_{c}\left(u,v,\theta \right)=\{\begin{array}{c} P_{0}\left(u,v,\theta \right)\;\;\;\;\;\;\;\;\;\;\;\;\;\;\;\;\;\;\;\;\;\;\;\;\;\;\;\;\;\;\;\;\;\;\;\;\;\;\;\;\;\;\;P_{0}\left(u,v,\theta \right)\leq t_{*}\\ \psi_{\lambda ,t_{*}}\left(P_{0}\left(u,v,\theta \right)\right)\;\;\;\;\;\;\;\;\;\;\;\;\;\;\;\;\;\;\;\;\;\;\;\;\;\;\;\;\;\;\;P_{0}\left(u,v,\theta \right)>t_{*} \end{array}$$

in which *u* and *v* represent the horizontal and vertical axes on the projection plane and *θ* is the scan angle of the CT scan based on a 2D X-ray detector, so-called a flat panel detector (FPD). As can be seen from Equation (1), the mapping is made for the projection data whose value exceeds the predetermined threshold $t_{*}$. The optimal mapping function has been found to be a combination of the exponential function $h_{\lambda ,t_{*}}$ and polynomials, as shown in Equation (2):

(2) $$\psi_{\lambda ,t_{*}}\left(P_{o}\left(u,v,\theta \right)\right)=h_{\lambda ,t_{*}}\left(u,v,\theta \right)+\overset{N}{\underset{n=2}{\sum }}\lambda_{n}{\left(P_{o}\left(u,v,\theta \right)-t_{*}\right)}^{n}$$

in which $h_{\lambda ,t_{*}}$ is the exponential function given below:

(3) $$h_{\lambda ,t_{*}}\left(u,v,\theta \right)=\frac{\lambda t_{*}-1}{2\lambda e^{-\lambda t_{*}}}e^{-\lambda P_{o}\left(u,v,\theta \right)}+\frac{\lambda t_{*}+1}{2\lambda e^{\lambda t_{*}}}e^{\lambda P_{o}\left(u,v,\theta \right)}$$

The threshold $t_{*}$ was experimentally determined to be the average value of projections passing through only the non-metallic regions.

**Figure 1:**
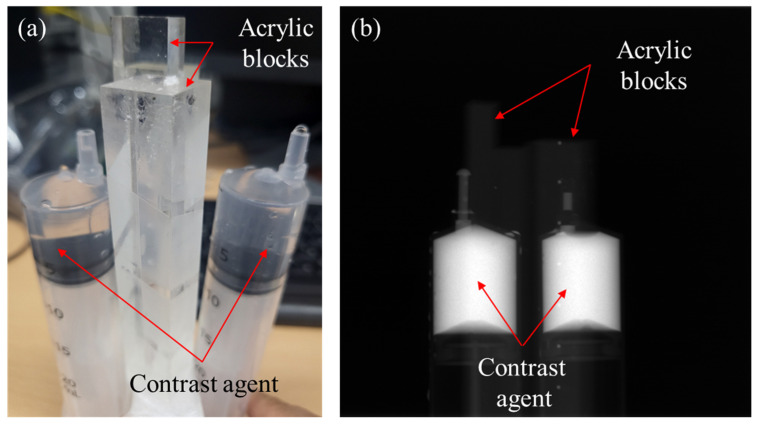
The contrast agent phantom consisting of two syringes filled with iodine solution (IOHEXOL) and two acrylic blocks. (**a**) A photographic image of the phantom and (**b**) a projection image of the contrast agent phantom.

From the experiments with applying the DSC method to the dental CT images of phantoms and patients, we observed that the DSC method works well when the metal artifacts are moderate. Yet, from the images with heavy metal artifacts, usually caused by multiple metallic objects, we often observed under-corrected metal artifacts with new streak artifacts appearing around the metallic objects. To investigate this problem, we made experiments with a phantom consisting of two syringes of a 25 mm diameter filled with iodine solution (IOHEXOL, GE Healthcare Ireland, Carrigtohill, Ireland) and two acrylic blocks, as shown in Figure 1a. We acquired three different projection data sets with three different iodine concentrations 300 mgI/mL, 150 mgI/mL, and 75 mgI/mL. An example of the projection data of the phantom with 300 mgI/mL concentration is shown in Figure 1b.

**Figure 2:**
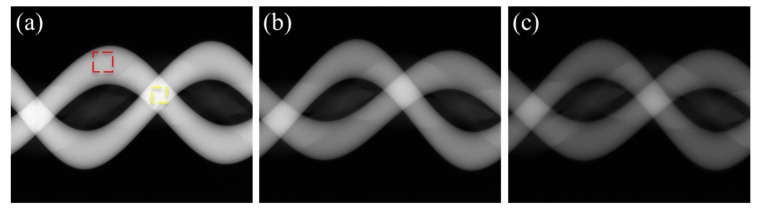
The sinograms made from the uncorrected projection data of the phantom with iodine concentrations of (**a**) 300 mgI/mL, (**b**) 150 mgI/mL, and (**c**) 75 mgI/mL, respectively.

**Figure 3:**
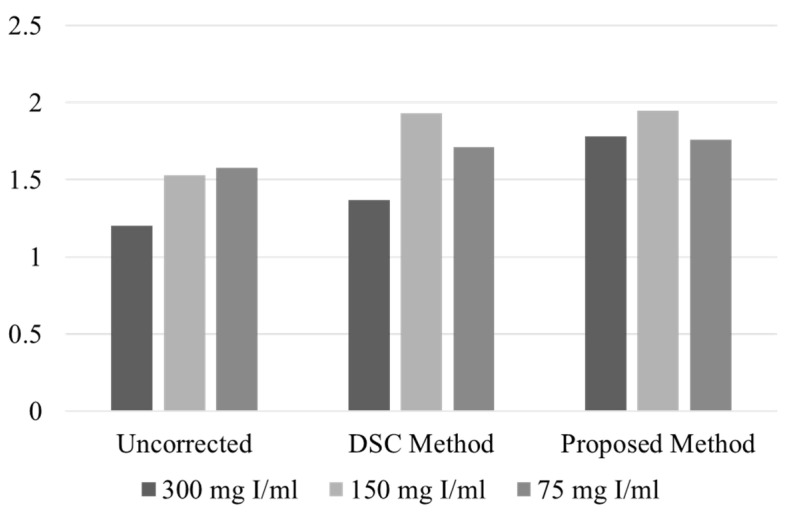
The projection intensity ratio of the yellow ROI to the red ROI shown in Figure 2.

Figure 2a–c show the original sinograms of the phantom with the iodine concentrations of 300 mgI/mL, 150 mgI/mL, and 75 mgI/mL, respectively. We measured the average projection intensity at two different regions of interest (ROIs): the yellow ROI on the overlapping iodine trace and the red ROI on the non-overlapping iodine trace, as shown in Figure 2a. We have summarized the average projection intensity and the average intensity ratio of the yellow ROI to the red ROI for the uncorrected and corrected sinograms in Table 1. The ratios are also compared in the graphs shown in Figure 3. Without the beam hardening effect, the average intensity ratio of the yellow ROI to the red ROI should be close to two since the path length of the iodine is doubled at the overlapped region. Yet, in the sinogram made from the original projection data, the ratio deviates from the ideal value of 2 because of the beam hardening effect and other limiting factors, such as X-ray scattering and nonlinear detector response. In the cases of low and middle concentrations (75 mgI/mL and 150 mgI/mL), the ratios (1.58 and 1.53) are similar to each other, but in the case of high concentration (300 mgI/mL), the ratio is greatly reduced to 1.20.

Using the DSC method, we corrected the sinograms of the phantom. With the mapping function utilizing only the *h* function in Equation (2), the optimization worked well for the low and middle concentrations, yielding the λ values of 0.315 and 0.329, respectively. In the corrected sinograms, the intensity ratio was recovered to 1.70 and 1.93 for the low and middle concentrations, respectively. Yet, in the case of high concentration, the optimization failed to converge. Therefore, we manually adjusted the λ value in such a way that the beam hardening effect was least visible in the reconstructed CT image. In that case, the λ value was 0.350 and the ratio was 1.37, which is still quite low compared to other concentrations.

**Figure 4:**
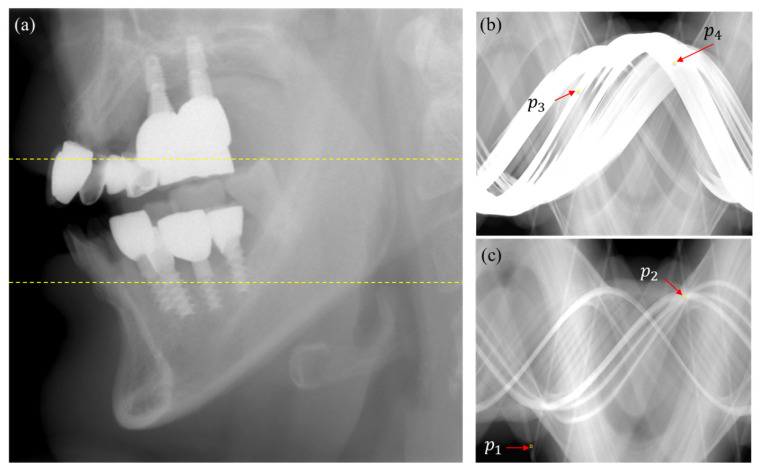
(**a**) An example of the projection image of a patient who has multiple dental implants and crowns. (**b**) A DSC sinogram made from the slice of the top dashed line. (**c**) A DSC sinogram made from the slice of the bottom dashed line.

We also observed similar behaviors from the projection data of patients having multiple metallic objects, such as dental implants, crowns, or dental fillings. Figure 4a shows an example of an uncorrected projection image that has multiple dental implants and dental crowns. Figure 4b,c show two sinograms corrected by the DSC method at the slices indicated by the upper and lower yellow lines in Figure 4a, respectively. On the metal trace of the sinogram (Figure 4c), the average projection intensity at the 5× 5 ROI around ($p_{1}$) on a non-overlapped metal trace is 0.52, while the average projection intensity at the ROI around ($p_{2}$), on which three metal traces are overlapped, is 3.47. The average projection intensity around $p_{2}$ is much higher than $p_{1}$, as expected from the increased metal path length of the ray hitting $p_{2}$. On the other hand, the average projection intensity around $p_{3}$ on the non-overlapped metal trace is 5.75, while the average projection intensity around $p_{4}$ on the overlapped metal trace is 4.62. Even though the projection at $p_{3}$ may have a bigger bone path length than at $p_{4}$, the lower intensity at $p_{4}$ is very unlikely. This observation also suggests that the DSC method would not work properly for patient images having multiple metallic objects.

The DSC method basically stretches up the projection data to a higher value if the projection data is on the metal trace. Our observation from the phantom and patient projection data suggest that the correction made by the DSC method would not be enough if the projection passes through multiple metallic objects, i.e., if the beam hardening effect is severe.

### 2.2. Modification of the Direct Sinogram Correction Method

**Figure 5:**
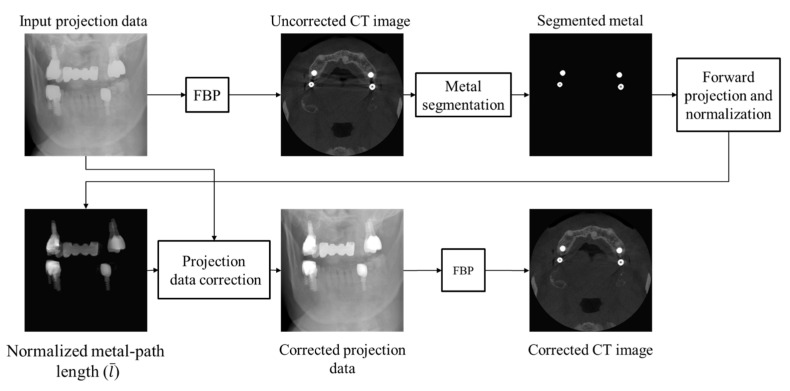
The workflow of the proposed metal artifact reduction method.

To alleviate the under-correction problem of the DSC method, we modified the mapping function employing the metal path-length weighting. Based on the observation from the experiments, we presume that the projection data mapping, working as projection data stretching, results in an under-correction of the metal artifacts and that the under-correction becomes more severe as the metal path length increases. To make bigger stretching for the projection data passing through metallic objects, we first segment the metallic objects in the uncorrected CT image using the segmentation method described in the next section. After segmenting the metallic objects in the CT image, we forward project the segmented metal mask to identify the metal trace on the sinogram with computing the metal path length of the ray hitting the metal trace. Then, we normalize the metal path length by the maximum value found all over the 3D projection data. We then use the normalized path length, $\bar{l}$, to stretch the projection data as follows:

(4) $$P_{c}\left(u,v,\theta \right)=\{\begin{array}{c} P_{o}\left(u,v,\theta \right)\;\;\;\;\;\;\;\;\;\;\;\;\;\;\;\;\;\;\;\;\;\;\;\;\;\;\;\bar{l}\left(u,v,\theta \right)\leq \varepsilon \\ H\left(P_{o}\left(u,v,\theta \right)\right)\;\;\;\;\;\;\;\;\;\;\;\;\;\;\;\;\;\;\;\;\;\bar{l}\left(u,v,\theta \right)>\varepsilon \end{array}$$

(5) $$H\left(P_{o}\left(u,v,\theta \right)\right)=\frac{-1}{2\lambda \prime }e^{-\lambda^{\prime }P_{o}\left(u,v,\theta \right)}+\frac{1}{2\lambda \prime }e^{\lambda^{\prime }P_{o}\left(u,v,\theta \right)}$$

(6) $$\lambda^{\prime }=\lambda \left(\bar{l}+\varepsilon \right)$$

where $P_{o}$ is the original projection data. ε is set to a small number so that the projection data passing through non-metallic regions will be little changed. We used ε = 10^−4^ in our experiments. Equation (5) is equivalent to Equation (2) if $t_{*}$ is set to zero. Since metal artifacts come from high-valued projection data, setting $t_{*}$ to zero has little effect on the metal artifact correction. When $P_{o}$ is large, as in the case of metallic projection data, the second term in Equation (5) dominates over the first term; hence, a higher projection data will be stretched more than a lower projection data. The parameter λ is now replaced by the metal path length-weighted $\lambda^{\prime }$. By multiplying the parameter λ by $\bar{l}$, we make a bigger stretching for the projection passing through a longer metal path. An optimal λ value can be found by optimizing the objective function used in the DSC method. If optimization fails, as frequently occurred in patient images, we manually find an optimal λ value from evaluating the metal artifact reduction in the reconstructed CT images. After mapping the whole 3D projection data, we then use the corrected projection data for the image reconstruction using the filtered back projection (FBP) method. The workflow of the proposed method is summarized in Figure 5.

### 2.3. Metal Segmentation

**Figure 6:**
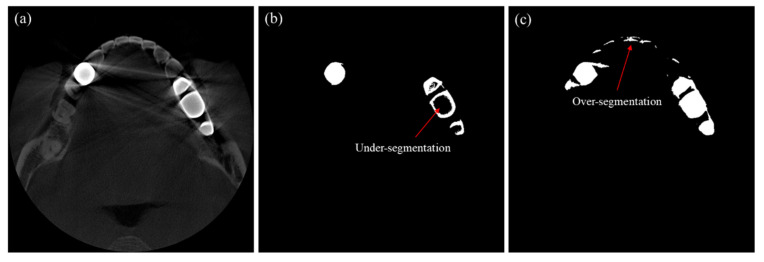
Examples of segmentation error when global thresholding is used for the segmentation. (**a**) The CT image to be segmented. (**b**) The segmentation result when a high threshold is applied. (**c**) The segmentation result when a low threshold is applied.

Metal segmentation is necessary to identify the metal trace on the sinogram. In segmenting an image having severe metal artifacts, applying a global threshold to identify the high-intensity metallic region may lead to erroneous segmentation. Figure 6b,c show examples segmented by the global thresholding method for the CT image shown in Figure 6a. When we applied a high threshold (4460 HU), the metallic objects were under-segmented, as shown in Figure 6b. On the other hand, when we applied a low threshold (2950 HU), all the metallic objects were included in the segment, but many non-metallic objects were wrongfully segmented, as shown in Figure 6c.

**Figure 7:**
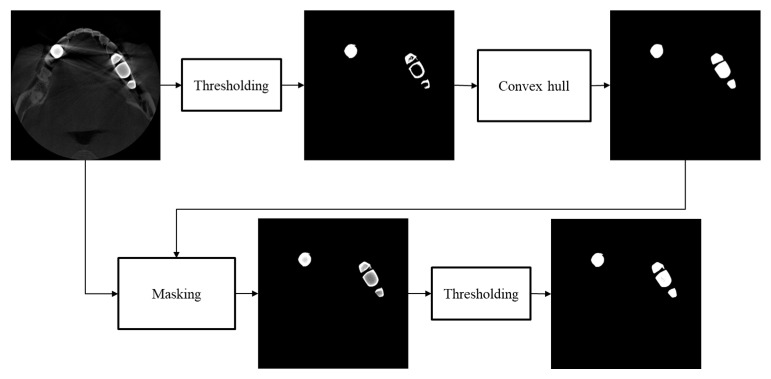
The workflow of the metal segmentation.

To better segment the metallic region in a metal artifact corrupted image, we first find an upper threshold that identifies only metallic regions without erroneously segmenting bone or tooth regions. With this threshold, we often see under-segmentation, i.e., hollows or wrong concaves in the metal mask, as can be seen in Figure 6b. To compensate the under-segmentation, we use the convex hull algorithm, which makes the smallest convex polygon that contains all the points in the under-segmented region [38,39,40]. Since the convex hull algorithm may include too many points outside the metallic region, we mask the original CT image using the convex hull mask, and we apply additional thresholding to the masked region with a lower threshold than that of the previous step. With this convex hull masking, we can improve the segmentation accuracy to the extent that small segmentation errors do not have any significant effects on the performance of metal artifact reduction. The upper and lower threshold we used for the patient cases were 4460 and 2950, respectively. The workflow of the metal segmentation is shown in Figure 7.

## 3. Results

**Figure 8:**
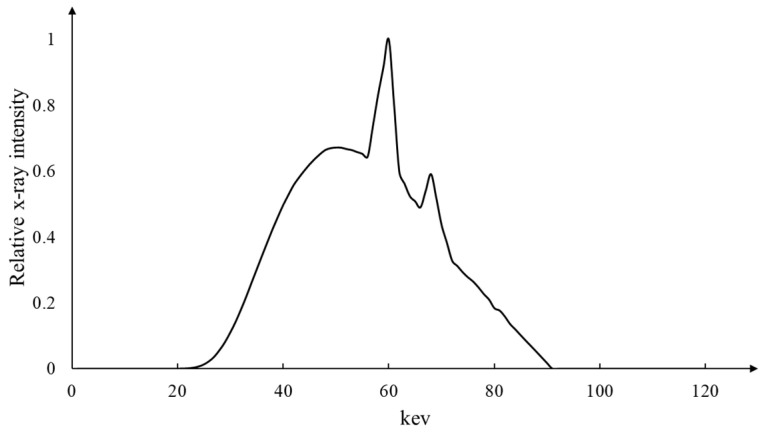
The X-ray spectrum emitted from the X-ray tube of the dental CT scanner (RAYSCAN α+, Ray, Republic of Korea).

To test the proposed method, we obtained some patient projection data acquired by a commercial dental CT scanner (RAYSCAN α+, Ray, Seongnam-si, Republic of Korea) with the tube voltage and current of 90 kVp and 10 mA, respectively. According to the institute’s ethical guidelines, we obtained the projection data from the dental clinics after removing the patient information. The X-ray spectrum emitted from the X-ray tube is shown in Figure 8. The anode of the X-ray tube is made of tungsten, and the tube voltage ripple was less than 1%. For X-ray filtration, we used a 0.2 mm thick copper filter in combination with a 2 mm thick aluminum filter. The inherent filtration of the X-ray tube was 0.8 mm thick aluminum. The dental scanner was equipped with a flat-panel detector with a matrix size of 584 × 584 and a pixel pitch of 248 µm. The detector bandwidth was 40~150 keV. The projection data were acquired with a number of projection views of 720 over 360 degrees. From the projection data, we reconstructed CT images with a matrix size of 626 × 626 × 626 and a pixel size of 0.16 × 0.16 mm^2^ using the Feldkamp algorithm [41].

We first applied the proposed mapping function to the projection data of the contrast agent phantom shown in Figure 2. Unlike the DSC method, the parameter λ of the mapping function was well optimized for all three concentration cases. The optimized λ values were 0.227, 0.248, and 0.318 for the low, middle, and high concentrations, respectively. As can be seen from Figure 3 and Table 1, the projection intensity ratios for the three concentrations, 1.76~1.95, are now more uniform as compared to the DSC method. In particular, the ratio for the high concentration case is much increased to 1.78, which implies better beam hardening correction in the CT image reconstruction. In the cases of low and middle concentrations, the beam hardening effect is not severe and both methods show similar correction of the projection data.

**Figure 9:**
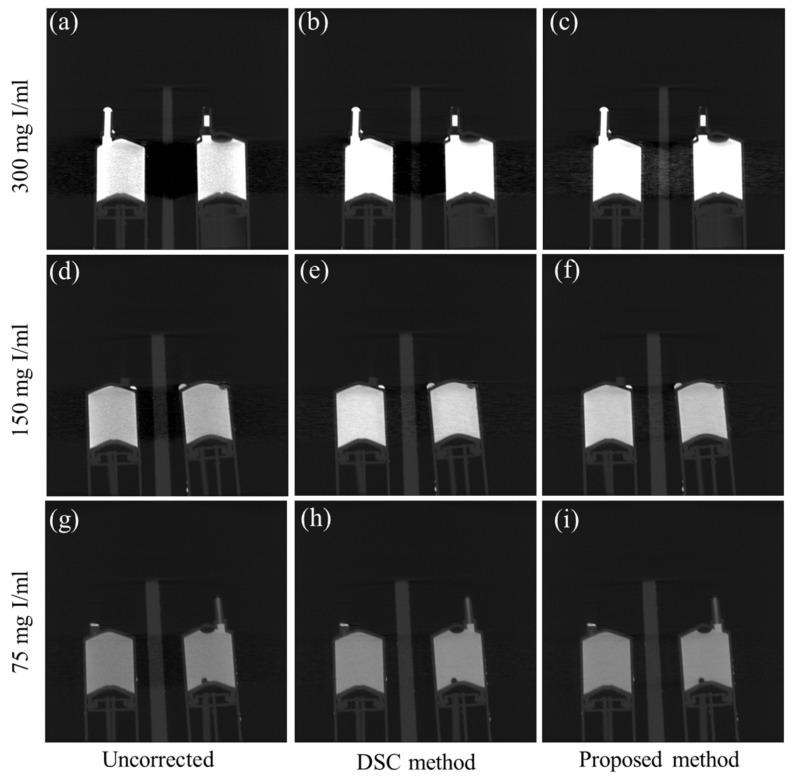
Examples of the beam hardening artifact reduction for the contrast agent phantom. The sagittal view images of the 300 mgI/mL phantom reconstructed from (**a**) the uncorrected projection data, and from the data corrected (**b**) by the original DSC method, and (**c**) by the proposed method, respectively. (**d**–**f**) The corresponding images of the 150 mgI/mL phantom. (**g**–**i**) The corresponding images of the 75 mgI/mL phantom. (Contrast window is [−1803~6972] in Hounsfield Unit).

Figure 9 shows the sagittal-view CT images of the contrast agent phantom. Figure 9a–c show the images of the high concentration phantom (300 mgI/mL) reconstructed from the uncorrected projection data, and the projection data corrected by the DSC method and the proposed method, respectively. We can easily notice that the beam hardening artifact is so severe in the uncorrected image that the acrylic structure placed in between the contrast agent tubes is completely invisible. In the image corrected by the DSC method, the acrylic structure is still quite invisible due to the under-correction of the beam hardening effect. However, in the case of the proposed method, the beam hardening artifacts are greatly reduced, and the acrylic structure is now clearly visible. On the other hand, when the beam hardening artifacts are small or moderate, as in Figure 9d,g, the DSC method (Figure 9e,h) and the proposed method (Figure 9f,i) show similar performance of beam hardening correction.

We also tested the proposed method on the CT images of patients having multiple dental implants, crowns, or dental fillings. For the patient images, both the DSC method and the proposed method failed to optimize the parameter λ, and we attribute it to the projection data truncation on the detector. The truncation was inevitable considering the limited detector width of 145 mm. From our experience with many types of adult patient images acquired with the tube voltage of 90 kVp, the λ value should be adjusted in the range between 0.6 and 0.9 depending on the number, size, and proximity of the metallic objects. If a patient has one or two small-sized metallic objects, the λ value is around 0.6, and the λ value increases up to 0.9 as the number and size of the metallic objects increase.

**Figure 10:**
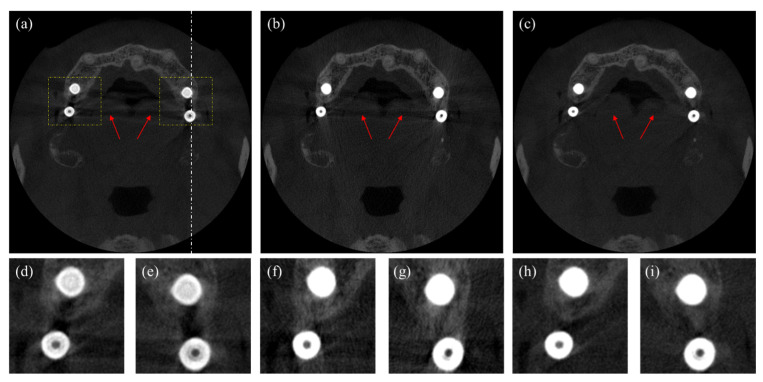
Axial images of a patient having four dental implants. (**a**) The uncorrected CT image, (**b**) the CT image corrected by the DSC method, and (**c**) the CT image corrected by the proposed method. The magnified images of the ROIs in (**d**,**e**) Figure 10a, (**f**,**g**) Figure 10b, and (**h**,**i**) Figure 10c. (Contrast window is [−1117~8564] in Hounsfield Unit).

In Figure 10, we compare images corrected by the DSC method and the proposed method. Figure 10a shows the uncorrected CT image, in which we can notice beam hardening-induced dark shadings in between the metallic dental implants and long shadings in the middle of the image, as indicated by the arrows. Figure 10b,c show the CT images corrected by the DSC method and the proposed method, respectively. We can see that the dark shadings in between the dental implants are corrected in both methods, but the long shadings in the middle of images still persist in the image corrected by the DSC method. In the image corrected by the proposed method, the dark shadings and the long shadings are almost invisible. In the bottom row in Figure 10, two magnified images of the ROIs in the upper image are shown, respectively. In Figure 10d,e, we can see that the dark shadings and streaks severely distort the surrounding bone structures. Yet, in Figure 10f,g, the dark shadings are greatly reduced, but the bone structures are not clearly seen due to the persisting streaks. In Figure 10h,i corrected by the proposed method, we can see that both the shading streak artifacts are greatly reduced, and the bone structures are better seen as compared to the image corrected by the DSC method.

**Figure 11:**
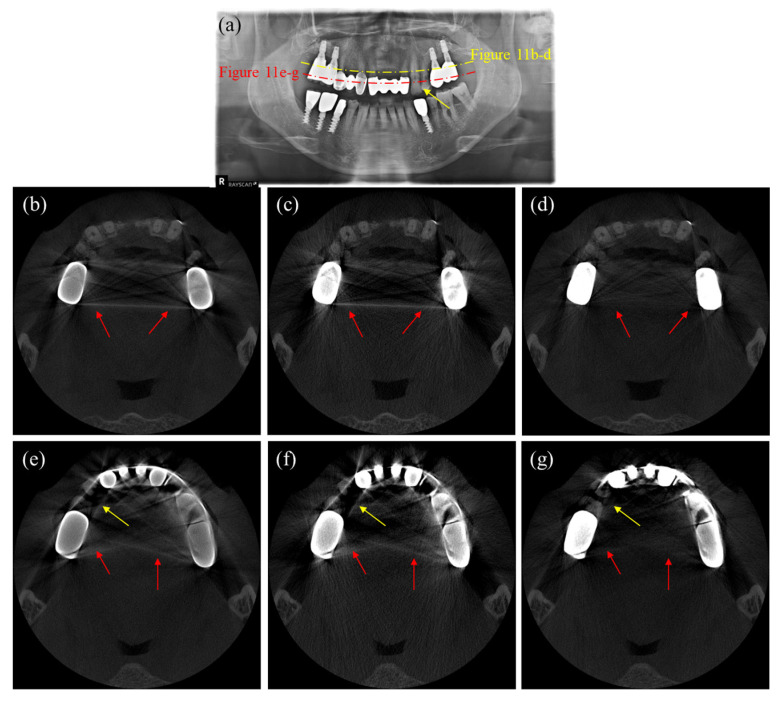
Images of a patient having multiple dental implants and crowns. (**a**) The panoramic X-ray image, (**b**,**e**) the uncorrected CT images, (**c**,**f**) the CT images corrected by the DSC method, and (**d**,**g**) the CT images corrected by the proposed method. (Contrast window is [−1117~8564] in Hounsfield Unit).

**Figure 12:**
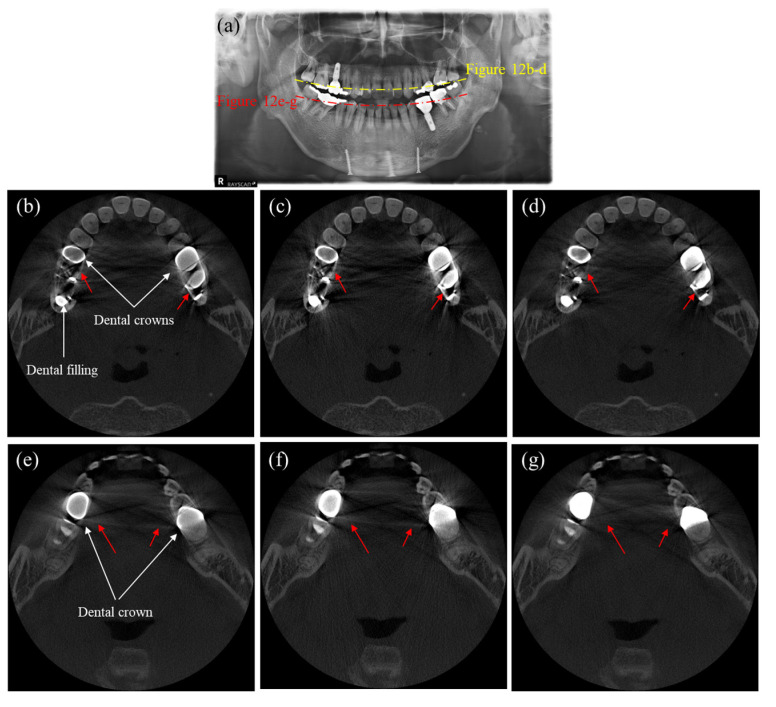
Images of a patient having multiple dental fillings and dental implants. (**a**) The panoramic X-ray image, (**b**,**e**) the uncorrected CT images, (**c**,**f**) the CT images corrected by the DSC method, and (**d**,**g**) the CT images corrected by the proposed method. (Contrast window is [−1117~8564] in Hounsfield Unit).

Figure 11 shows axial CT and panoramic X-ray images of a patient having multiple dental implants and crowns. Since we cannot have an artifact-free CT image as a reference, we use the panoramic X-ray image, shown in Figure 11a, to evaluate the MAR performance. Figure 11b–d are the CT images corresponding to the yellow dashed line in Figure 11a, while Figure 11e–g are the CT images corresponding to the red dashed line. As can be noticed from the uncorrected CT images shown in Figure 10a and Figure 11b, the metal artifacts in Figure 11 are more severe than in Figure 10 due to the big-sized crowns positioned near each other. In this case, the DSC method fails to reduce the metal artifacts, as shown in Figure 11c,f. However, the images corrected by the proposed method show a great reduction of metal artifacts, as shown in Figure 11d,g. The two intact teeth in between the crowns, as indicated by the yellow arrows in the panoramic image, are not visible in Figure 11e,f, but they are visible in Figure 11g, even though they are somewhat distorted. Similarly, Figure 12 shows the images of another patient having multiple dental implants and dental fillings. Figure 12a is the panoramic X-ray image, and Figure 12b–d are the CT images corresponding to the yellow dashed line, while Figure 12e–g are the CT images corresponding to the red dashed line in the panoramic image. Figure 11e and Figure 12b show the uncorrected CT images, while Figure 12c,f show the images corrected by the DSC method. After correction by the DSC method, we can see that shading and streaks are still persisting. Figure 12d,g show the images corrected by the proposed method. Again, the proposed method better reduced the shadings and streaks than the DSC method.

**Figure 13:**
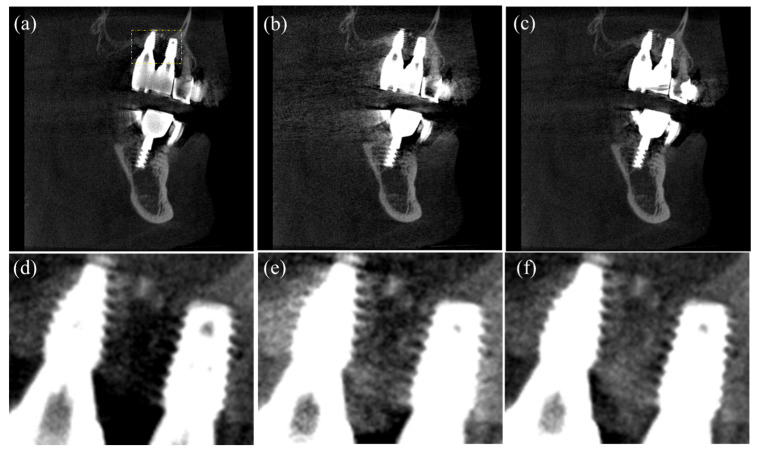
Sagittal-view images of the patient shown in Figure 10. (**a**) The uncorrected image. (**b**) The image corrected by the DSC method. (**c**) The image corrected by the proposed method. (**d**–**f**) The corresponding zoomed image at the ROI shown in Figure 13a. (Contrast window is [−625~5083] in Hounsfield Unit).

Figure 13 shows the sagittal-view images of the patient shown in Figure 10. The line of the sagittal slice is indicated by the white dashed line in Figure 10a. Figure 13a shows the uncorrected CT images, while Figure 13b,c show the images corrected by the DSC method and the proposed method, respectively. Figure 13d–f show the corresponding zoomed images at the ROI indicated by the yellow rectangle in Figure 13a. In the zoomed images, we can see that both the DSC and proposed methods have recovered well the bone structures surrounding the implant screws.

## 4. Discussion

Most dental CT systems use flat-panel detectors (FPDs) for X-ray detection. Even though FPDs have a few advantages over discrete detectors, such as two-dimensional image acquisition capability with very fine pixel pitches, low cost, and compact size, FPDs have lower sensitivity to the X-ray exposure, and they have higher spatial non-uniformity of the detector response over the pixels. The responsivity of a pixel element of an FPD may vary over the time depending on the X-ray exposure and the detector temperature, and some of the pixel elements may lose responsivity to the extent that the pixel elements become dead. Those weak FPD characteristics have negative effects on the image quality; hence, the image quality of dental CTs is known to be poorer than that of medical CTs. Apart from the weak features of FPDs, dental CT systems have other limitations, such as low tube voltage/current and slow scan speed. Low tube voltage, usually under 100 kV, may cause too much attenuation of the X-rays through the metallic objects. Hence, more severe metal artifacts may appear in the dental CT images than in the images taken by a medical CT with higher tube voltage.

In our experiments, we observed that the original DSC method does not work well for patients who have multiple dental implants, crowns, or dental fillings. There will be many reasons for the malfunctioning, and X-ray scattering, and dark current drift are among them. Since most dental CT systems do not use grids to block the scattered X-rays, dental CT images are more prone to scattering artifacts. From the patient images having many metallic objects, we often observed that the projection intensity on the metal trace after the correction was smaller than what was expected from the correction. The original DSC method considered only the polychromatic attenuation caused by the photo-electric effect and Compton scattering in deriving the mapping function. Unlike the beam hardening artifacts appearing as shadings and streaks, the scatter effect appears as edge blurring and CT-number anomaly on the CT images [42]. In the sinogram correction, any projection intensity change caused by the scattered X-rays will cause erroneous mapping of the projection data, hence leading to either under-correction or over-correction of the metal artifacts. Therefore, we need to separate the scatter-induced intensity change from the beam hardening-induced intensity change. We think the scatter effect has been partially compensated by applying the metal path length weighting to the DSC method. Offset drifts of the FPD output can also have negative effects on the correction performance. Offset drifts caused by the dark current fluctuation of the FPD will change the projection data greatly when the X-ray intensity on a detector pixel is very low due to the heavy X-ray attenuation. Since the sinogram is made by taking the logarithm of the X-ray intensity I, a small change in I made by the offset drift can make a big change in the sinogram due to the rapid slope of the logarithm function at a small argument near zero. It is also known that the offset may drift in a large amount over the temperature and X-ray exposure to the FPD [43,44,45]. It means that the offset effects may vary over the scan time.

The optimization to find the parameter λ worked well for the phantom images, in which there was no truncation of the projection data on the small-sized detector. Yet, the optimization failed in the case of patient images. Truncation of the projection data makes spatially variant CT-number anomaly [46,47], hence any optimization based on the CT image formation will be affected by the truncation. We think the optimization failure in the case of patient images was due to the truncation effect and the X-ray scatter. Considering the wide width of jaw bones and their high X-ray attenuation and scatter behaviors, the truncation and scatter effects on the patient images will be much higher than on the phantom images. However, finding an optimal λ value manually was not a difficult job since the response of artifact reduction to λ was found to be a good convex. We could find an optimal λ after a few trials. It would be practical to find optimal λ values in advance for a few categories of patient images, depending on the number, size, and proximity of metallic objects, and use them later for clinical imaging.

The proposed metal segmentation method needs two thresholds, the upper and lower thresholds in the two-step segmentation procedure. The segmentation performance will depend on the selection of these thresholds, but for most patient data acquired with the same CT scan parameters, the thresholds may be fixed and they do not need to be adjusted for every patient. In our study, we used the same thresholds for all the patient cases, which did not affect the MAR performance. We believe we can use the same thresholds for other patients having different teeth and metal configurations, but it should be verified by further extensive studies.

## 5. Conclusions

We proposed a modified DSC method to reduce severe metal artifacts, and we tested it on human dental CT images. By applying bigger weighting to the projection passing through a longer metal path length in the sinogram correction, we could make improvements in the metal artifact reduction. The proposed method is based on an intuitive modification of the DSC method, and it lacks rigorous mathematical backgrounds. Therefore, we need to have more extensive studies to verify the clinical applicability of the proposed method with many more patient images obtained with various CT scan conditions.

## Figures and Tables

**Table 1:** The projection intensity at the ROI and the projection intensity ratio of the yellow ROI to the red ROI.

	Uncorrected			DSC Method			Proposed Method		
Iodine concentration (mgI/mL)	300	150	75	300	150	75	300	150	75
Average intensity in the yellow ROI (A)	6.14	5.24	4.15	8.73	7.16	4.80	10.01	6.67	4.73
Average intensity in the red ROI (B)	5.13	3.43	2.63	6.37	3.71	2.80	5.63	3.42	2.68
Intensity ratio (A/B)	1.20	1.53	1.58	1.37	1.93	1.70	1.78	1.95	1.76

## Data Availability

Data is unavailable due to privacy and ethical restrictions.
